# Remotely supervised at-home tDCS for veterans with persistent post-traumatic headache: a double-blind, sham-controlled randomized pilot clinical trial

**DOI:** 10.3389/fneur.2023.1184056

**Published:** 2023-05-05

**Authors:** Leigh Charvet, Adam T. Harrison, Kiersten Mangold, Robert Davis Moore, Siyuan Guo, Jiajia Zhang, Abhishek Datta, X. Michelle Androulakis

**Affiliations:** ^1^Department of Neurology, New York University Langone Health, New York, NY, United States; ^2^Arnold School of Public Health, University of South Carolina, Columbia, SC, United States; ^3^Department of Neurology, Columbia VA Healthcare System, Columbia, SC, United States; ^4^Department of Biostatistics & Bioinformatics, Duke University School of Medicine, Durham, NC, United States; ^5^Research and Development, Soterix Medical, Inc., Woodbridge, NJ, United States; ^6^Department of Biomedical Engineering, City College of New York, New York, NY, United States; ^7^Headache Centers of Excellence Program, US Department of Veterans Affairs, Columbia, SC, United States

**Keywords:** veterans, tDCS, mTBI, post-traumatic headache, brain injury

## Abstract

**Background:**

Currently, there are no FDA approved therapies for persistent post-traumatic headache (PPTH) secondary to traumatic brain injury (TBI). As such neither headache nor TBI specialists have an effective means to manage PPTH. Thus, the objective of the present pilot trial was to evaluate the feasibility and preliminary efficacy of a four-week at-home remotely supervised transcranial direct current stimulation (RS-tDCS) intervention for veterans with PPTH.

**Methods:**

Twenty-five (*m* = 46.6 ± 8.7 years) veterans with PPTH were randomized into two groups and received either active (*n* = 12) or sham (*n* = 13) RS-tDCS, with anodal stimulation over left dlPFC and cathodal over occipital pole. Following a four-week baseline, participants completed 20–sessions of active or sham RS-tDCS with real-time video monitoring over a period of four-weeks. Participants were assessed again at the end of the intervention and at four-weeks post-intervention. Primary outcomes were overall adherence rate (feasibility) and change in moderate-to-severe headache days per month (efficacy). Secondary outcomes were changes in total number of headache days, and PPTH-related functional outcomes.

**Results:**

Adherence rate was high with 88% of participants (active = 10/12; sham = 12/13) fully completing tDCS interventions. Importantly, there was no significant difference in adherence between active and sham groups (*p* = 0.59). Moderate-to-severe headache days were significantly reduced within the active RS-tDCS group (*p* = 0.004), compared to sham during treatment (−2.5 ± 3.5 vs. 2.3 ± 3.4), and 4-week follow-up (−3.9 ± 6.4 vs. 1.2 ± 6.5). Total number of headache days was significantly reduced within the active RS-tDCS (*p* = 0.03), compared to sham during-treatment (−4.0 ± 5.2 vs. 1.5 ± 3.8), and 4-week follow-up (−2.1 ± 7.2 vs. −0.2 ± 4.4).

**Conclusion:**

The current results indicate our RS-tDCS paradigm provides a safe and effective means for reducing the severity and number of headache days in veterans with PPTH. High treatment adherence rate and the remote nature of our paradigm indicate RS-tDCS may be a feasible means to reduce PPTH, especially for veterans with limited access to medical facilities.

Clinical Trial Registration: ClinicalTrials.gov, identifier [NCT04012853].

## 1. Introduction

Persistent post-traumatic headache (PPTH) is one of the most common types of chronic pain conditions experienced among veterans with traumatic brain injury (TBI) (1, 2). PPTH is characterized as a chronic headache disorder lasting more than three months and is 1) a secondary headache disorder that develops or 2) a worsening primary headache disorder, in close temporal relation to a TBI (3). The prevalence and incidence of TBI and PPTH have increased dramatically in veterans, especially in those returning from Operation Enduring Freedom and Operation Iraqi Freedom (4). Although PPTH frequently manifests as migraine or chronic migraine, many patients fail to respond to conventional migraine therapies (5). Currently there is no FDA approved treatment for PPTH and the escalating opioid crisis raises concerns about medication overuse and abuse in this population. Co-existing PPTH with polytrauma triad (TBI, chronic pain, and PTSD) further complicates functional recovery and overall quality of life. Our research demonstrates veterans with comorbid TBI and persistent headaches is associated with a greater risk of suicide attempts than other types of chronic pain (6). Therefore, there is a critical need to identify and provide effective treatment for veterans with PPTH.

Transcranial direct current stimulation (tDCS), a safe and well-tolerated non-invasive brain stimulation technique, utilizes continuous, low-intensity direct electrical current to modulate resting membrane potential (7, 8). Although the exact effect of tDCS on neuronal behavior is largely unknown, it is believed to facilitate or inhibit neuronal firing rate by generating sub-threshold depolarization or hyperpolarization, depending on direction of current flow (7). In addition to the direct impact on the neuronal resting membrane potential, tDCS is also believed to elicit changes in neurotransmitter release, neuroinflammatory processes, as well as cerebrovascular behavior (9–11). Although the acute effects of tDCS only last approximately 1 hour, repeated sessions can produce cumulative and long-lasting modulations of neural activity and neuroplasticity (12, 13). Unsurprisingly, tDCS has gained attention as a potential therapeutic tool for use in a range of various neuropsychiatric conditions (14–20). Our systematic review and meta-analysis of tDCS for migraine found that repeated tDCS sessions can significantly reduce headache intensity and duration (21). However, to our knowledge, there are no randomized, sham-controlled, double-blind clinical trials published on the feasibility and efficacy of at-home remotely supervised tDCS (RS-tDCS) for with PPTH.

Until recently the clinical implementation of tDCS has been limited by logistical factors, however, modern tDCS devices are portable, programmable, and easy to operate. Furthermore, tDCS can be delivered to patients at home via telehealth applications with real-time clinical monitoring (22). Previous research has demonstrated that RS-tDCS interventions are feasible and effective in a number of clinical populations such as Parkinson’s Disease, Multiple Sclerosis, Alzheimer’s Disease, Stroke, and TBI (17, 23–26). At-home delivery of RS-tDCS offers an accessible and appealing option for veterans who may not be able to travel to clinics for regular treatments. Accordingly, our primary objectives were to evaluate the feasibility and preliminary efficacy of a four-week RS-tDCS intervention using real-time video monitoring in veterans with PPTH.

We conducted a randomized, double-blinded, sham-controlled pilot clinical trial comparing active RS-tDCS with anodal stimulation over left dlPFC and cathodal stimulation over occipital pole vs. sham RS-tDCS. More specifically, we compared the efficacy of 20-sessions of 20-min active, 2 mA anodal vs. sham RS-tDCS. Our primary outcome measures were adherence rate, and reduction in number of moderate-to-severe headache days during the intervention as well as four-weeks post-intervention. Our secondary and tertiary outcome variables were changes in total number of headache days, and headache-related disability during the intervention as well as four-week post-intervention. We hypothesized that our RS-tDCS intervention would have high adherence rates (greater than 80%) (23), and that individuals receiving active RS-tDCS at would report significant reductions in number of moderate-to-severe headache days, total number of headache days and headache related disability during treatment and at four-week follow-up compared to sham RS-tDCS.

## 2. Methods

The study procedures for this pilot randomized sham-controlled clinical trial (ClinicalTrials.gov Identifier: NCT04012853) were approved by Department of Veterans Affairs (VA) institutional review board in Columbia, South Carolina. All participants provided written informed consent prior to enrollment and were eligible for financial compensation. This study followed the Consolidated Standards of Reporting Trials (CONSORT) guidelines (27).

### 2.1. Study population

**Figure 1:**
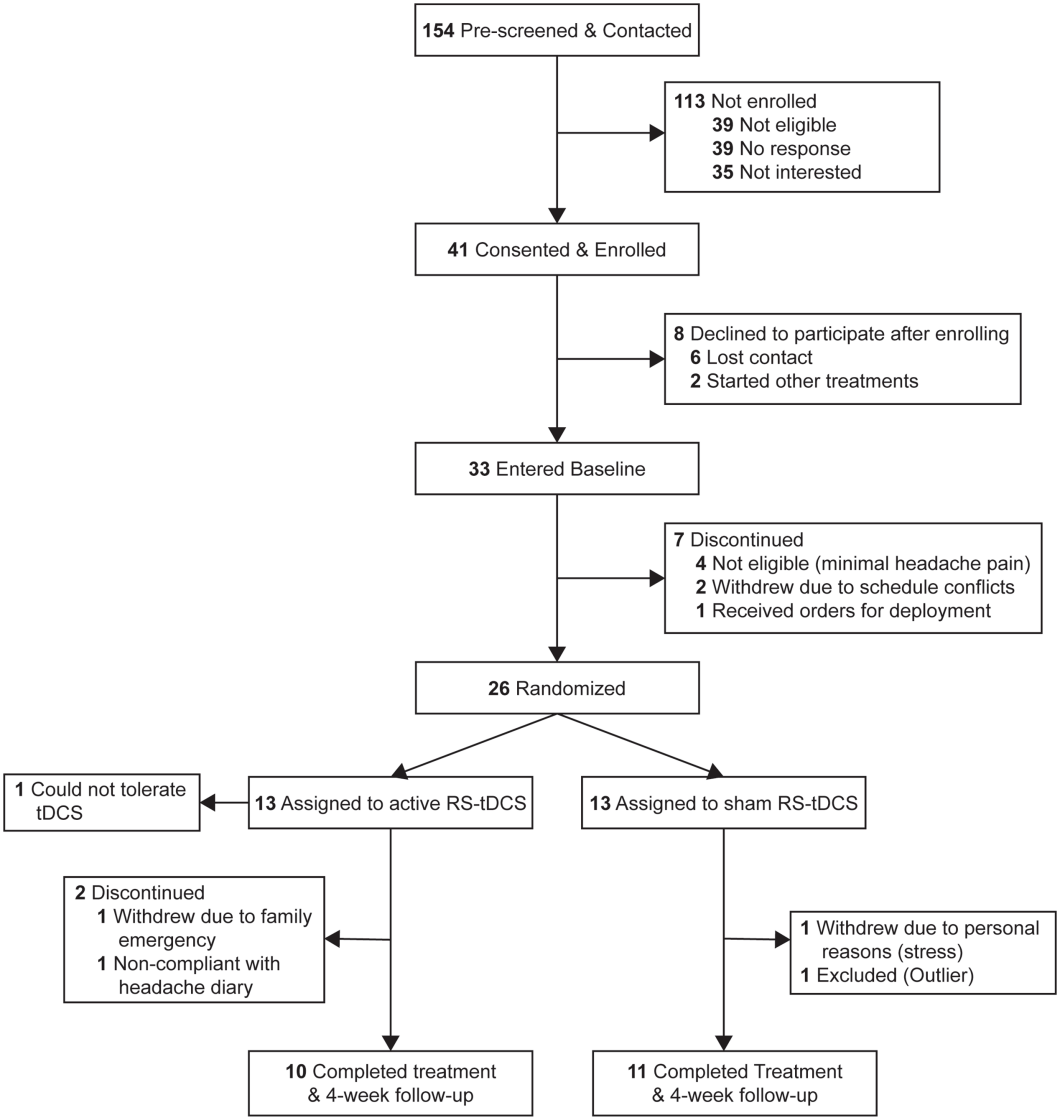
CONSORT flow diagram.

Participants were recruited through the Columbia VA Medical Center. Identified patients were contacted and pre-screened by members of the research team. Figure 1 illustrates recruitment and enrollment of included participants. All participants were active or retired military service members between the ages of 20–60 years (*m* = 46.6 ± 8.7 years) with a verified mTBI, and who met the International Classification of Headache Disorders (ICHD III) diagnostic criteria for “persistent headache attributed to traumatic head injury” (28). Prior to randomization, enrolled participants were asked to complete a 28-day baseline headache diary to confirm headache characteristics/inclusion criteria.

### 2.2. Study design

**Figure 2:**
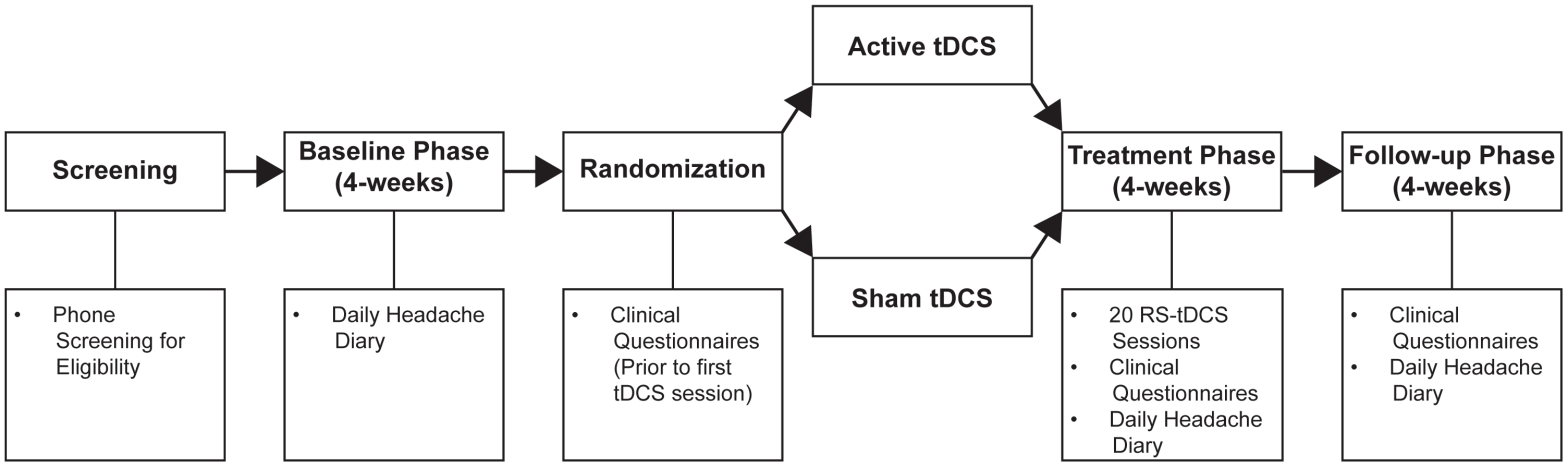
RS-tDCS intervention and treatment timeline.

This randomized, double-blind, sham-controlled, pilot clinical trial consisted of an initial screening and recruitment phase, baseline observation phase (four-weeks), treatment phase (four-weeks), and post-treatment follow-up (four-weeks; Figure 2). During the baseline phase, participants who met inclusion and exclusion criteria (Supplementary material) were invited to complete a four-week headache diary. This was followed by an in-person introductory RS-tDCS training session and a tDCS stimulation tolerability test. A member of the study team provided initial training and ensured that each participant would be able to operate the equipment at home. Participants then completed the tDCS tolerability test to ensure that they could comfortably tolerate the tDCS stimulation. For the tolerability test, tDCS intensity was gradually ramped up to the target intensity of 2 mA. Participants were asked whether the intensity was tolerable and were prompted to report any adverse reactions. Participants were excluded if they did not tolerate the target stimulation intensity. Participants completed the first tDCS session in-person immediately following their tolerability test and the remaining 19-sessions were completed at home with remote supervision via VA Telehealth Video Connect (VVC).

Prior to the in-person tDCS session participants were randomized into either active or sham RS-tDCS conditions and completed a series of questionnaires (Figure 2). These questionnaires encompassed headache-related disability (Headache Impact Test; HIT-6), depression (Patient Health Questionnaire; PHQ-9), PTSD-related symptoms (DSM-5 PTSD Checklist; PCL-5), anxiety (Beck’s Anxiety Inventory; BAI), sleep disturbances (Insomnia Severity Index; ISI), and post-concussive symptoms (Rivermead Post-Concussive Symptoms Questionnaire; RPQ). Participants repeated these questionnaires at the end of their respective intervention and at 4-week post-treatment follow-up.

#### 2.2.1. Randomization and RS-tDCS stimulation protocol

Participants were randomized (1:1) using a random number generator (R Studio v3.4.1, Boston, MA). To maintain double blinding, a clinic nurse who is not part of the study team pre-programmed each device according to their group randomization assignment.

**Figure 3:**
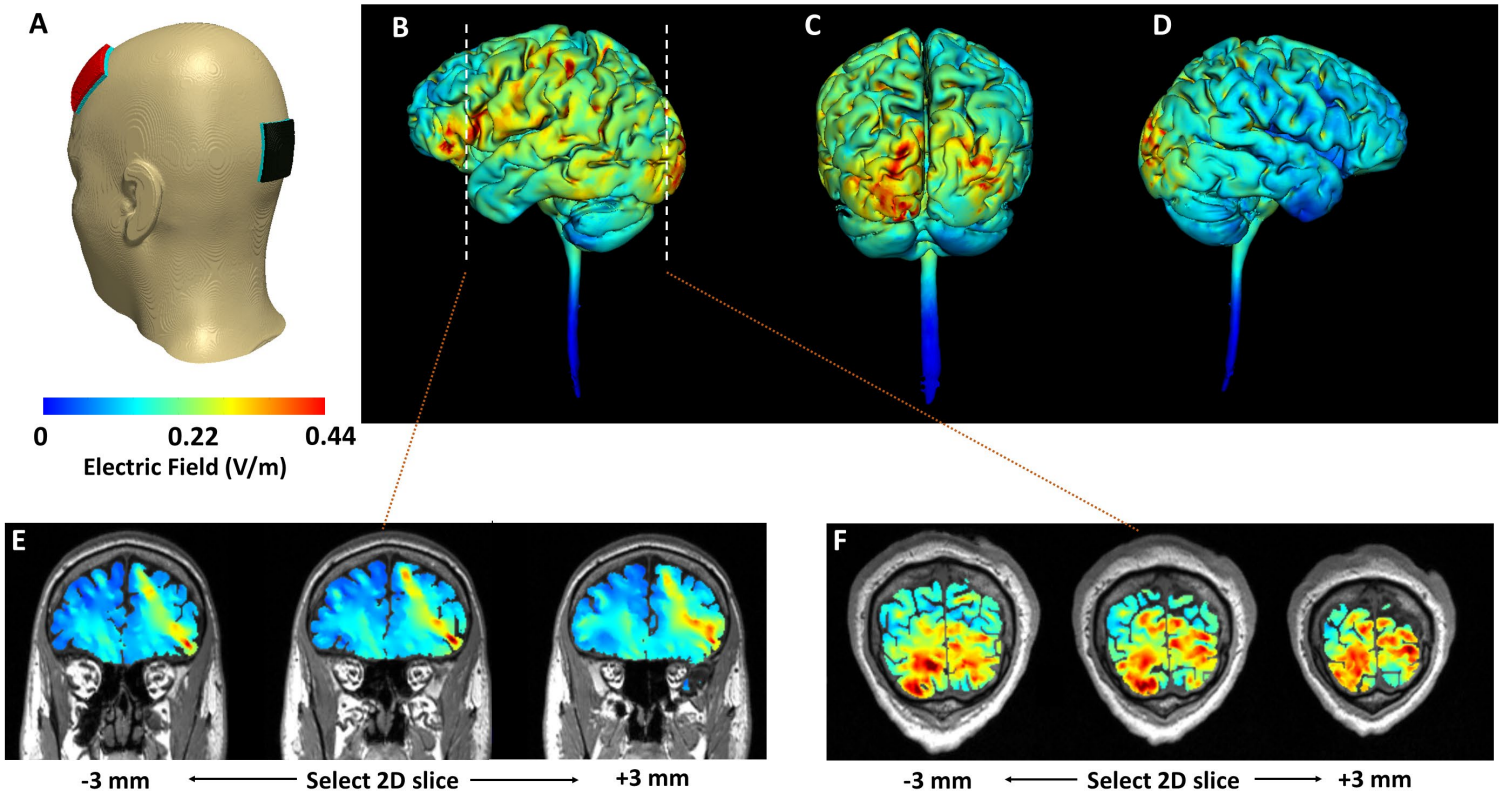
Computational simulation of electric field distribution. **(A)** Model geometry considered. Red electrode indicates placement of the anode (F3). Black electrode indicates placement of the cathode (Oz). Cortical surface (3D) plots are included in **(B–D)**. Cortical cross-sectional (2D) plots that highlight depth focality/flow in deeper subcortical regions are included in **(E,F)**. **(B)** Left lateral view. **(C)** Posterior view. **(D)** Right lateral view. The corresponding cross-sectional slices from the left dorsolateral prefrontal cortex and the occipital cortex (dashed line in **B**) are shown in **(E,F)**, respectively. Slices at +/−3 mm from the selected 2D slice are also plotted to further highlight induced current flow patterns.

**Figure 4:**
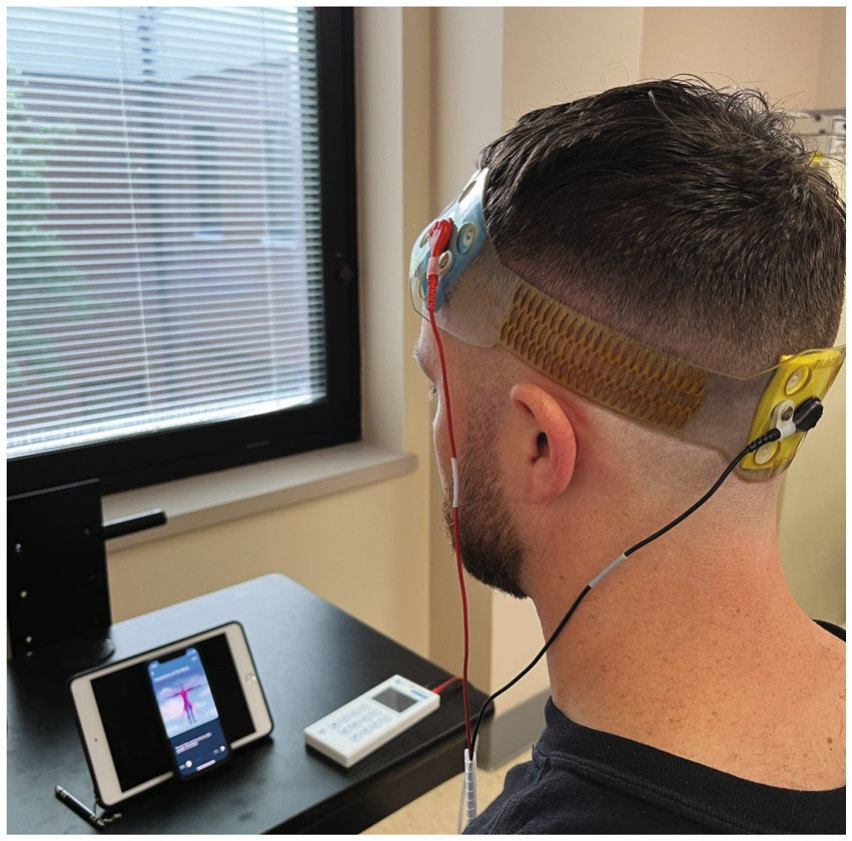
RS-tDCS montage.

To ensure consistent electrode placement, each head strap was configured according to the international 10–20 system (29). We used our novel stimulation montage, based on computational modeling (see Figure 3) with the anodal electrode placed over the left dlPFC (F3) and the cathode placed over the occipital pole (Oz). Stimulation was delivered via the FDA approved for investigational use Mini-CT (Soterix Medical, Woodbridge, NJ); and patients were given a study kit consisting of customized headgear with electrodes, sponges, rechargeable batteries, and battery charger. The unique electrodes (SNAPpad) allow loading onto headgear (SNAPstrap) at fixed locations preventing incorrect electrode placement (see Figure 4).

We developed this novel electrode montage as both mTBI and migraine are linked to abnormal functional connectivity within frontal brain regions such as the left dlPFC (30–33). Previous research demonstrates that anodal stimulation over the left dlPFC is more effective than M1 for migraine (34). However, most studies utilize a reference (cathode) electrode placed over the supraorbital region. Because emotional reactivity and mood disturbance are common in mTBI and PPTH patients (35–38), we wanted to avoid inadvertently modulating cortical regions involved in behavioral and mood regulation (e.g., right PFC, inferior frontal gyrus) located near SO. Finally, migraine patients often demonstrate greater neuronal excitability within the occipital lobe (39), and cathodal stimulation over the lower occipital pole has shown to be effective in pain reduction in migraine (40).

Each at-home session was monitored by a member of the study team via HIPAA compliant VVC. At the beginning of each session, participants were given a code by the supervising researcher to unlock the tDCS device. Each tDCS device’s stimulation parameters were uniquely programmed and could only be unlocked with a one-time code. For active tDCS, stimulation was gradually increased during the first 30-s to the target intensity (2 mA) and maintained for the remainder of the session (19-min) and then ramped down gradually during the last 30-s. For sham tDCS, the device was programmed to gradually ramp up to 2 mA and back down to zero in both the first and last minute of the session, with no current being delivered in between (41). All RS-tDCS sessions were paired with mindfulness meditation to serve as an attentional control consistent across participants and sessions. The meditation sessions were identical each day and consisted of voice-guided mindfulness exercises designed to promote awareness of body and breathing via the VA Mindfulness Coach app (US Department of Veterans Affairs). If participants missed treatment sessions during the week, they were allowed to “make up” the session on weekend or by extending the intervention timeline to a 5th week.

#### 2.2.2. Headache diary

A headache diary was used to capture individual headache characteristics throughout the duration of each four-week (28-days) phase of the intervention (baseline, treatment, and post-treatment). The headache diary was adapted from the VA Cognitive Behavioral Therapy for Headache manual (42). Due to variability in how individuals describe pain we adopted the following scale: mild (nagging, annoying headache with little to no interference with daily activity), moderate (headache that is bothersome, interferes significantly with daily activity, and usually requires medication), and severe (disabling or intolerable pain that causes inability to perform routine daily activity).

### 2.3. Clinical outcomes

#### 2.3.1. Primary outcomes

Feasibility was defined by the participant’s completion of ≥16 sessions (80% adherence) (43). Adherence rate and participant discontinuations were characterized and compared between the active and sham RS-tDCS conditions.

Efficacy was evaluated by comparing changes in number of moderate-to-severe headache days from the baseline to the end of treatment and at their four-week follow-up evaluation.

#### 2.3.2. Secondary outcomes

Change in total number of headache days, headache disability (HIT-6), and days of acute pain medication use from the baseline to the end of treatment and at their four-week follow-up evaluation.

#### 2.3.3. Tertiary outcomes

Change in depressive symptoms (PHQ-9), anxiety symptoms (BAI), PTSD-related symptoms (PCL-5), sleep disturbance (ISI), and post-concussive symptoms (RPQ) from the baseline phase to the intervention phase and follow-up phase.

#### 2.3.4. Intervention related side effects/tolerability

Participants were asked to report any perceived treatment related side-effects at the end of each RS-tDCS session.

### 2.4. Statistical analyses

Independent two-tailed t-tests and Fisher Exact tests were used to compare continuous and categorical (respectively) group level demographics, TBI characteristics, and baseline headache features. To investigate measures of safety and tolerability, as well as feasibility and compliance, we compared group differences in self-reported treatment side-effects and intervention attrition using a series of Fisher Exact tests. To assess the efficacy of our RS-tDCS intervention change scores relative to baseline (Evaluation – Baseline) were computed for as well as tertiary outcomes and secondary outcome measures collected at each post-intervention evaluation. Normality was assessed using Shapiro–Wilk’s test of normality and homogeneity of variance was assessed by Levene’s test. Group outliers were identified as individual values that exceeded ±2.5 standard deviations at each evaluation timepoint for both primary and secondary outcome measures. Participants were one participant was excluded as he was consistently identified as an outlier across primary and secondary outcomes from further analyses if they were consistently identified as an outlier across primary and secondary outcomes. Next, a series of group (SHAM, ACTIVE) × time (Post-Treatment, Follow-up) univariate analysis of variance (ANOVA) were computed for each outcome measure. All statistical analyses were conducted with an *a priori* alpha = 0.05. For significant interactions and main effects, Bonferroni corrections for multiple comparisons were applied.

## 3. Results

### 3.1. Participants

From December 2019 to March 2022, 154 patients were screened for eligibility. Forty-one participants who met inclusion and exclusion criteria were consented and enrolled in the study (Figure 1). Thirty-three participants completed baseline headache diaries, and 26 eligible participants were randomly assigned to either active (*n* = 13) or sham (*n* = 13) RS-tDCS treatment. One participant was unable to tolerate the target intensity and never began treatment. Twenty-two participants completed the full intervention (active *n* = 10; sham *n* = 12). One participant was determined to be an outlier and was removed leaving a total of 21 participants (active *n* = 10; sham *n* = 11) in the final analysis (see Supplementary Figure S1). There were no significant differences in demographics, or baseline TBI and clinical characteristic between groups (*p*’s ≥ 0.08; Table 1). Participants were predominantly white (12/21), and male (18/21), adults and 10 of the 21 participants had at least some college or technical school education. There was no significant difference in the number of days to complete needed to complete the RS-tDCS treatment among SHAM (*m =* 29.3 ± 2.5 days) or active (*m =* 28.9 ± 1.7 days) groups (*p* = 0.7). Medication use was similar between groups and no participant was taking opioids or benzodiazepines (Supplementary Table S3).

**Table 1:** Baseline demographics and headache characteristics.

	Active RS-tDCS (*n* = 10)	Sham RS-tDCS (*n* = 11)	*p*-value
Age (years)	49.3 ± 8.5	42.6 ± 8.0	0.08
Sex (# men)	9 (90%)	9 (81.8%)	1.00
Body mass tndex (kg/m^2)	28.9 ± 4.9	29.9 ± 4.9	0.64
Race (*n*)			0.67
White	5 (50%)	7 (63.6%)	
Black or African American	5 (50%)	4 (36.4%)	
Marital Status (*n*)			0.82
Married	7 (70%)	8 (72.7%)	
Divorced	2 (20%)	2 (18.2%)	
Never married/domestic partnership	1 (10%)	1 (9.1%)	
Education Level (*n*)			0.61
High School graduate or GED	2 (20%)	1 (9.1%)	
Some college or technical school	4 (40%)	7 (63.6%)	
Bachelor’s degree or higher	4 (40%)	3 (27.3%)	
Employment Status (*n*)			0.24
Employed (full or part time)	4 (40%)	7 (66.6%)	
Unemployed	3 (30%)	0	
Disabled	1 (10%)	2 (18.2%)	
Retired	2 (20%)	2 (18.2%)	
TBI characteristics			
Number of injuries	2.4 ± 1.0	2.3 ± 1.1	0.54
Years since first injury	16.6 ± 9.2	15.3 ± 9.8	0.75
TBI mechanism (*n*)^a^			
Blast	5	6	1.00
Mortar	3	2	0.62
Motor vehicle accident	4	3	0.65
Other	5	6	1.00
Headache characteristics			
Age at headache onset (years)	30.7 ± 9.2	30.6 ± 9.3	0.96
Number of headache days (out of 28 days)	25.6 ± 3.8	24.1 ± 5.0	0.45
Number of moderate to severe headache days (out of 28 days)	15.6 ± 8.8	15.7 ± 7.1	0.97
Headache phenotype			1.00
Migraine-like	9 (90%)	9 (81.8%)	
Tension-type	1 (10%)	2 (18.2%)	
Acute pain medication use (days out of 28)	11.5 ± 11.3	9.5 ± 8.1	0.64
^b^Medication overuse (*n*)	4 (40%)	4 (36.4%)	1.00
Quality of life			
PHQ-9	14.3 ± 6.8	14.1 ± 5.0	0.94
HIT-6	64.5 ± 6.7	65.3 ± 6.1	0.79
BAI	30.6 ± 15.0	26.5 ± 15.4	0.54
PCL-5	47.6 ± 21.6	44.6 ± 19.3	0.74
ISI	18.1 ± 8.7	18.6 ± 5.9	0.87
RPQ	44.7 ± 14.6	43.5 ± 9.1	0.82

a Not mutually exclusive. Values reported as mean ± standard deviation unless otherwise indicated. GED, General Educational Development Test; TBI, Traumatic brain injury; PHQ-9, Patient Health Questionnaire; HIT-6, Headache Impact Test; BAI, Beck Anxiety Inventory; PCL-5, DSM-5 PTSD Checklist; ISI, Insomnia Severity Index; RPQ, Rivermead Post-Concussion Symptoms Questionnaire.

b Medication overuse is defined as taking one acute pain medication more than 15 days/month or 2+ acute pain medications more than 10 days/month.

### 3.2. Safety and tolerability

Supplementary Figure S2 shows the reported side-effects by treatment group. As expected, most side-effects reported were related to mild sensations at the electrode site. The three most common side-effects were: 1) tingling (active 70%/sham 91.7%), 2) warm sensation (active 30%/sham 58.3%), and 3) itching (active 20%/sham 25%). There were no significant differences in side-effect reporting between the active vs. sham RS-tDCS groups (*p*’s > 0.08). Side-effects diminished shortly after the end of each training session, with no lasting side-effects reported by any participant. Importantly, no participant reported any side-effects or adverse events that required discontinuation of the treatment session or withdrawal from the intervention.

### 3.3. Primary outcomes

#### 3.3.1. Feasibility

Eighty-eight percent (22/25) completed the intervention (active 10/12 vs. sham 12/13, Figure 1), and there was no significant difference in adherence rate between groups (*p* = 0.59).

#### 3.3.2. Moderate-to-severe headache days

**Figure 5:**
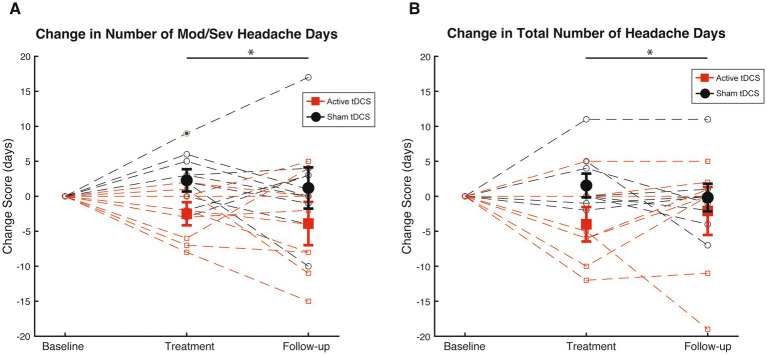
**(A)** Mean [SE] changes in moderate-to-severe headache days (Mod/Sev HA days) during treatment phase and follow-up among active RS-tDCS (red square) and sham (black circle) groups. **(B)** Mean [SE] changes in total headache days (HA days) during treatment phase and follow-up among active RS-tDCS (red square) and sham (black circle) groups. * Indicates a group difference at *p*<0.05.

Figure 5A illustrates individual and group changes in moderate-to-severe headache days over the course of the RS-tDCS intervention. Omnibus analysis of changes in moderate-to-severe headache days revealed an effect for group (*F*_[1,38]_ *=* 9.2, *p* = 0.004, *η*^2^ = 0.19), with the active RS-tDCS group reporting a greater reduction in moderate-to-severe headache days during the intervention and at 4-week follow-up (est. mean = −3.2 ± 1.2) compared to sham (est. mean = 1.7 ± 1.1). No other significant differences were observed (Table 2).

**Table 2:** Change in primary and secondary outcomes among active vs. sham RS-tDCS groups.

				Effect size (*η*^2^) [*p*-value]		
	Group	Treatment	Follow-up	Interaction	Group	Time
Primary Outcome						
Mod/Sev HA Days (*n*)	sham	2.3 ± 3.4	1.2 ± 6.5	<0.01 [0.92]	**0.19 [<0.01]**	0.01 [0.45]
	active	−2.5 ± 3.5	−3.9 ± 6.4			
Secondary Outcomes						
HA Days (*n*)	sham	1.5 ± 3.8	−0.2 ± 4.4	0.03 [0.27]	**0.12 [0.03]**	<0.01[0.96]
	active	−4.0 ± 5.2	−2.1 ± 7.2			
Tertiary Outcomes						
PHQ-9	sham	0.1 ± 4.4	−1.0 ± 5.6	0.01 [0.54]	<0.01[0.96]	<0.01[0.89]
	active	−0.6 ± 4.1	0.1 ± 4.2			
HIT-6	sham	0.8 ± 5.4	−5.4 ± 18.4	0.03 [0.33]	0.01 [0.57]	0.02 [0.41]
	active	−4.5 ± 6.5	−4.0 ± 7.3			
BAI	sham	1.9 ± 7.4	3.5 ± 10.8	<0.01[0.84]	0.10 [0.06]	<0.01[0.73]
	active	−3.2 ± 8.6	−2.8 ± 9.6			
PCL-5	sham	1.9 ± 10.5	−1.4 ± 17.2	<0.01[0.89]	<0.01[0.91]	0.03 [0.35]
	active	2.9 ± 9.3	−1.5 ± 12.4			
RVMD	sham	−4.2 ± 11.7	−4.0 ± 14.8	0.01 [0.57]	0.01 [0.46]	0.01 [0.54]
	active	−10.2 ± 19.1	−4.8 ± 10.6			
ISI	sham	0.2 ± 5.1	0.9 ± 8.4	<0.01[0.85]	0.01 [0.58]	<0.01[0.85]
	active	−0.5 ± 5.5	−0.5 ± 4.2			

Mod/Sev HA Days, Moderate-to-Severe Headache days; HA Days, Headache Days; PHQ-9, Patient Health Questionnaire; HIT-6, Headache Impact Test; BAI, Beck Anxiety Inventory; PCL-5, DSM-5 PTSD Checklist; ISI, Insomnia Severity Index; RPQ, Rivermead Post-Concussion Symptoms Questionnaire. Bold values indicate statistical significance at *p*<0.05.

### 3.4. Secondary outcomes

#### 3.4.1. Secondary headache outcomes

Figure 5B omnibus analysis of reduction in headache days revealed an effect for group (*F*_[1,38]_ *=* 5.3, *p* = 0.03, *η*^2^ = 0.12), indicating that irrespective of timepoint, the active RS-tDCS group reported a greater reduction in total headache days (est. mean = −3.0 ± 1.2) compared to sham (est. mean = 0.7 ± 1.1). No significant reductions were observed for headache-related disability or acute pain medication usage (*p*’s ≥ 0.57). No other significant differences were observed (Table 2).

### 3.5. Tertiary outcomes

#### 3.5.1. PPTH-related functional outcomes

Omnibus analyses failed to detect any significant group or time differences in non-headache-related outcome measures (*p*’s ≥ 0.06) among active or sham RS-tDCS groups (see Table 2). However, it should be noted that the active group exhibited a statistical trend towards reduced anxiety across timepoints (*p* = 0.06, *η*^2^ = 0.10).

## 4. Discussion

Veterans with PPTH frequently present with migraine or chronic migraine phenotypes (82% in our cohort) (44). However, typical migraine therapies are often ineffective for managing headaches or reducing PPTH-related disability (45). Furthermore, traditional therapeutic approaches for TBI are minimally effective for managing chronic pain in veterans with PPTH (24). Unsurprisingly, veterans with PPTH have disproportionately poor outcomes relative to their peers, including increased rates of joblessness, homelessness, and suicidality (6). This double-blind, randomized, sham-controlled, pilot clinical trial provides preliminary evidence for the feasibility and efficacy of a novel at-home RS-tDCS with real-time monitoring protocol for veterans with PPTH.

In our first of its kind clinical trial, we demonstrated that a four-week RS-tDCS intervention was feasible (high adherence rate), and well tolerated by veterans with PPTH. Specifically, 88% of veterans that began treatment completed the intervention in its entirety, and there was no significant difference between groups. This further validates findings from broader tDCS literature, demonstrating tDCS is well tolerated by a wide-range of clinical populations (19, 46–48). Most importantly, compared to sham stimulation, veterans receiving active stimulation reported decreases in moderate-to-severe headache days and total number of headache days both during the intervention and at four-week follow-up; providing the first evidence that RS-tDCS is an effective treatment for veterans with PPTH.

The exact mechanisms by which tDCS reduces pain is not fully understood. However, tDCS is believed to modulate excitatory neurotransmitter release, post-synaptic N-methyl-D-aspartate (NMDA) receptor over binding, neuroinflammation, and the synchronous activity of neurons, all of which are key pathophysiological factors underlying both chronic mTBI, migraine, and PPTH (11, 49–51). Prior research demonstrates tDCS improves neuronal synchronization and reduces hyper-excitability in veterans with chronic TBI (45, 46) and reduces spectral perturbations in those with migraine (47, 48). However, whether the changes occur in those with PPTH is unknown. Future research employing biological and psychophysiological measures will help elucidate tDCS mechanisms of change in those with PPTH and help guide future interventions.

It should be noted that although we observed significant changes in our primary and secondary outcomes, common comorbidities associated with mTBI and chronic headache such as depression, anxiety, post-traumatic stress, and insomnia were not significantly different. Although perplexing, this could be due to several factors including our novel montage, which was purposefully selected to target and related functional outcome (headache severity associated disability). Thus, our targeted montage configuration may have resulted, as intended, in pain specific neuromodulations.

However, it is also possible that the small sample size could account for current null results in tertiary outcomes. Changes in anxiety were nearly significant (*p* = 0.06) across timepoints and the observed effect sizes were moderate (*η*^2^ = 0.10). Given the extant literature on the efficacy of tDCS for modulating anxiety and its neural progenitors (44), it is possible with a larger sample size changes in anxiety would have been significant. However, research including more participants and longer duration interventions are necessary to gain a better understanding of the influence of our RS-tDCS protocol on anxiety in veterans with PPTH.

Together, our results indicate that a relatively short term (1 month) RS-tDCS intervention can result in significant changes in moderate-to-severe headache days and total number of headache days. Furthermore, these benefits were maintained one-month post-intervention, suggesting the benefit extend beyond the intervention. Therefore, patients may be able to cycle RS-tDCS therapies while maintaining efficacy. This is an important point as many patients undergoing tDCS find the prospect of long-term daily/regular stimulation as burdensome. Indeed, one of the current limitations of self-administrated neuromodulation therapy (e.g., Cefaly, Gammacore VNS) for headache is decreasing adherence rates over time (23) and being able to cycle neuromodulation therapies makes them a more realistic option from a patient and provider perspective. Additional research comparing various intervention lengths, stimulation intensities and duration are needed to create a standardized platform.

### 4.1. Limitations

Although our study is characterized by several strengths, there are limitations to consider. First, our final sample was relatively small and comprised of predominantly white, male military veterans. As such, our findings should not be generalized to female veterans, or non-military PPTH patients, and larger phase II clinical trials are necessary to confirm our findings. Second, tDCS is not FDA approved to treat PPTH or any other neurological condition. Accordingly, there are no guidelines for optimal parameter selection. Results of previous studies have been highly variable and direct comparisons are difficult due to these methodological differences. Also, parameters such as stimulation intensity and number of sessions are directly correlated to the duration of effects. While the parameters of our trial are consistent with existing literature, it is possible that more training sessions and/or higher stimulation intensities could result in greater benefits. Medication use is a key issue in clinical trials (52) and common preventative migraine medications (anticonvulsant, anti-depressants) are known to impact tDCS effects (53). Restricting medication use in veterans with mTBI with numerous comorbidities is often not clinically feasible. Consequently, we could not control for medication use in such a small trial. Fortunately, medication use was equally distributed between groups (Supplementary Table S3), and no participants were taking opioids or benzodiazepines. Future, more controlled research is necessary to determine the benefit of RS-tDCS independent of medication use. Finally, we had no true control group as all participants completed mindfulness meditation as an attentional control during their tDCS sessions. Although long-duration mindfulness meditation has shown to be an effective therapy for chronic migraine, the short durations have not (54). Furthermore, significant changes were only observed for active stimulation in both within and between groups analyses, suggesting mindfulness meditation did not significantly influence primary or secondary outcomes.

## 5. Conclusion

Our results indicate the combined feasibility and efficacy of RS-tDCS may provide a promising non-pharmacological alternative for veterans suffering from PPTH. Furthermore, having the option to conduct neuromodulation sessions remotely will greatly facilitate caring for veterans in more rural communities, where daily visits to medical facilities are impractical. Based on these promising preliminary results, larger clinical trials should be conducted to optimize the therapeutic benefit of RS-tDCS for veterans with PPTH secondary to mTBI. Furthermore, identifying the biological and psychophysiological changes that occur from RS-tDCS in this population is warranted.

## Data availability statement

The datasets presented in this article are not readily available because, due to VA regulations and Veterans Health Administration ethics agreements, the analytical datasets used for this study are not permitted to leave the VA firewall without data-use agreement. Requests to access the datasets should be directed to XA, xiao.androulakis@va.gov.

## Ethics statement

The studies involving human participants were reviewed and approved by U.S. Department of Veterans Affairs Institutional Review Board in Columbia, South Carolina. The patients/participants provided their written informed consent to participate in this study. Written informed consent was obtained from the individual for the publication of any potentially identifiable images or data included in this article.

## Author contributions

LC conceived the project, assisted in device training and protocol development, participated in the manuscript writing process read, and approved the final manuscript. AH, KM, SG, and JZ participated in data analysis and interpretation of results, participated in the manuscript writing process read, and approved the final manuscript. RM participated in data analysis and interpretation of results. AD assisted in device training and protocol development, participated in the manuscript writing process, read and approved the final manuscript. XA conceived the project, obtained IRB approval, participated in data analysis and interpretation of results, participated in the manuscript writing process, read and approved the final manuscript. All authors contributed to the article and approved the submitted version.

## Funding

Grant support for the trial was received from the US Department of Veterans Affairs Research and Development, Award number RX-003194-01A1. The funding agency played no part in the execution of the trial or the interpretation/reporting of results. XA received funding from the VA Headache Centers of Excellence.

## Conflict of interest

AD was employed by Soterix Medical, Inc.

The remaining authors declare that the research was conducted in the absence of any commercial or financial relationships that could be construed as a potential conflict of interest.

## Publisher’s note

All claims expressed in this article are solely those of the authors and do not necessarily represent those of their affiliated organizations, or those of the publisher, the editors and the reviewers. Any product that may be evaluated in this article, or claim that may be made by its manufacturer, is not guaranteed or endorsed by the publisher.
