# Supplementary material for: Remotely supervised at-home tDCS for veterans with persistent post-traumatic headache: a double-blind, sham-controlled randomized pilot clinical trial
Source: Front Neurol. 2023 May 5;14:1184056. doi: 10.3389/fneur.2023.1184056 (PMC10196360; doi:10.3389/fneur.2023.1184056)

*Inclusion criteria*:

1. Verified history of mTBI using VA TBI identification clinical interview screening criteria,^26^
2. Persistent PTH as defined by ICHD-III diagnostic criteria.^25^
3. Verification of headache frequency through prospectively collected baseline information during the 28-day screening/baseline phase.
4. Not currently taking a migraine or headache preventive medication OR has been taking a stable dose of a preventive for at least 60 days prior to screening and agrees to not start, stop, or change medication and/or dosage during the study period.
5. We will include medication overuse headache as defined by ICHD III diagnostic criteria.
6. Participants is either not of childbearing potential, or if they are of childbearing potential, they agree either to remain abstinent or use (or have their partner use) an acceptable method of birth control for the duration of the study.
7. Male or female, age between 20-60 who demonstrates compliance with Daily Headache Diary during the 28-day baseline phase as defined by entry of headache data on a minimum of 22 to 28 days (80% diary compliance).

*Exclusion criteria:*

1. Unable to complete headache diary as required by protocol.

2. Any psychiatric condition with psychotic features, and/or any other psychiatric disorder not stable or well controlled, that would interfere in the ability to complete study activities

3. Received botulinum toxin A, cognitive behavior therapy, physical therapy, or any other form of non-pharmacological therapy for headaches during the 4 months before screening.

4. Has a planned military deployment within the 6 months post screening.

5. Active substance abuse within last 4 months.

6. History of seizure, stroke, multiple sclerosis or other unstable neurological condition or a significant abnormal neurological examination.

7. Unable to tolerate 2mA tDCS stimulation.

8. Have any other conditions that in the judgment of the Investigator would make the participants unsuitable for inclusion or interfere with participating or completing the study.

**Supplemental Table 1.** Baseline Demographics and Headache Characteristics (Outlier Included)

|  | **Active RS-tDCS**  (n = 10) | **Sham**  **RS-tDCS**  (n = 12) | **Comparison**  (p-value) |
| --- | --- | --- | --- |
| **Age (years)** | 49.3 ± 8.5 | 43.3 ± 7.9 | 0.10 |
| **Sex (# men)** | 9 (90%) | 10 (83.3%) | 1.00 |
| **Body Mass Index (kg/m^2)** | 28.9 ± 4.9 | 29.65 ± 4.76 | 0.72 |
| **Race (n)** |  |  | 0.67 |
| White | 5 (50%) | 8 (66.7%) |  |
| Black or African American | 5 (50%) | 4 (33.3%) |  |
| **Marital Status (n)** |  |  | 0.82 |
| Married | 7 (70%) | 9 (75%) |  |
| Divorced | 2 (20%) | 2 (16.67%) |  |
| Never married/domestic partnership | 1 (10%) | 1 (8.33%) |  |
| **Education Level (n)** |  |  | 0.61 |
| High School graduate or GED | 2 (20%) | 1 (8.3%) |  |
| Some college or technical school | 4 (40%) | 7 (58.3%) |  |
| Bachelor's degree or higher | 4 (40%) | 4 (33.3%) |  |
| **Employment Status (n)** |  |  | 0.24 |
| Employed (full or part time) | 4 (40%) | 8 (66.67%) |  |
| Unemployed | 3 (30%) | 0 |  |
| Disabled | 1 (10%) | 2 (16.67%) |  |
| Retired | 2 (20%) | 2 (16.67%) |  |
| **TBI Characteristics** |  |  |  |
| Number of Injuries | 2.4 ± 1.0 | 2.1 ± 1.0 | 0.42 |
| Years Since First Injury | 16.6 ± 9.2 | 15.3 ± 9.8 | 0.75 |
| **TBI Mechanism (n)*** |  |  |  |
| Blast | 5 | 6 | 1.00 |

| Mortar | 3 | 2 | 0.62 |
| --- | --- | --- | --- |
| Motor Vehicle Accident | 4 | 3 | 0.65 |
| Other | 5 | 7 | 1.00 |
| **Headache Characteristics** |  |  |  |
| Age at Headache Onset (years) | 30.7 ± 9.2 | 30.6 ± 9.3 | 0.96 |
| Number of Headache Days (out of 28 days) | 25.6 ± 3.8 | 24.6 ± 4.1 | 0.56 |
| Number of Moderate to Severe Headache Days (out of 28 days) | 15.6 ± 8.8 | 15.9 ± 7.2 | 0.50 |
| Acute Pain Medication Use (days out of 28 | 11.5 ± 11.3 | 9.6 ± 7.9 | 0.65 |
| Medication Overuse (n) | 4 (40%) | 4 (33.3%) | 1.00 |
| **Quality of Life** |  |  |  |
| PHQ-9 | 14.3 ± 6.8 | 14.3 ± 4.6 | 0.99 |
| HIT-6 | 64.2 ± 6.4 | 63.2 ± 7.7 | 0.74 |
| BAI | 31.6 ± 16.2 | 22.1 ± 10.1 | 0.11 |
| PCL-5 | 46.2 ± 20.0 | 42.7 ± 19.2 | 0.68 |
| ISI | 17.8 ± 8.5 | 17.7 ± 6.7 | 0.97 |
| RPQ | 42.0 ± 13.03 | 42.0 ± 10.0 | 1.00 |

*Note: not mutually exclusive. Values reported as mean ± standard deviation unless otherwise indicated. GED: General Educational Development Test; TBI: Traumatic brain injury; PHQ-9: Patient Health Questionnaire; HIT-6: Headache Impact Test; BAI: Beck Anxiety Inventory; PCL-5: DSM-5 PTSD Checklist; ISI: Insomnia Severity Index; RPQ: Rivermead Post-Concussion Symptoms Questionnaire

**Supplemental Table 2**. Change in primary and secondary outcomes among active vs. sham RS-tDCS groups (Outlier Included)

|  |  |  |  | Effect Size (η^2^) [p-value] | | |
| --- | --- | --- | --- | --- | --- | --- |
|  | Group | Treatment | Follow-up | Interaction | Group | Time |
| ***Primary Outcome*** | | | | | | |
| Mod/Sev HA Days (n) | SHAM | 1.0±5.5 | -0.4±8.3 | <0.01 [0.99] | 0.08 [0.08] | 0.01 [0.47] |
|  | ACTIVE | -2.5±3.5 | -3.9±6.4 |  |  |  |
| ***Secondary Outcomes*** | | | | | | |
| HA Days (n) | SHAM | 0.6±4.9 | -1.5±6.2 | 0.03 [0.27] | 0.05 [0.16] | <0.01[0.96] |
|  | ACTIVE | -4.0±5.2 | -2.1±7.2 |  |  |  |
| PHQ-9 | SHAM | -0.6±4.8 | -1.6±5.7 | <0.01 [0.57] | <0.01[0.55] | <0.01[0.92] |
|  | ACTIVE | -0.6±4.1 | 0.1±4.2 |  |  |  |
| HIT-6 | SHAM | 1.3±5.3 | -5.0±17.5 | 0.03 [0.30] | 0.01 [0.47] | 0.02 [0.37] |
|  | ACTIVE | -4.5±6.5 | -4.0±7.3 |  |  |  |
| BAI | SHAM | 0.9±7.8 | 2.2±11.1 | <0.01[0.88] | 0.06 [0.12] | <0.01[0.77] |
|  | ACTIVE | -3.2±8.6 | -2.8±9.6 |  |  |  |
| PCL-5 | SHAM | 0.0±11.8 | -3.4±17.6 | <0.01[0.90] | <0.01[0.56] | 0.02 [0.35] |
|  | ACTIVE | 2.9±9.3 | -1.5±12.4 |  |  |  |
| RPQ | SHAM | -4.6±11.2 | -3.9±14.0 | <0.01 [0.59] | 0.01 [0.46] | 0.01 [0.48] |
|  | ACTIVE | -10.2±19.1 | -4.8±10.6 |  |  |  |
| ISI | SHAM | -0.4±5.2 | 0.3±8.8 | <0.01[0.98] | <0.01 [0.92] | <0.01[0.98] |
|  | ACTIVE | -0.5±5.5 | -0.5±4.2 |  |  |  |

Note:Mod/Sev HA Days: Moderate-to-severe headache days; HA Days: Headache days; PHQ-9: Patient Health Questionnaire; HIT-6: Headache Impact Test; BAI: Beck Anxiety Inventory; PCL-5: DSM-5 PTSD Checklist; ISI: Insomnia Severity Index; RPQ: Rivermead Post-Concussion Symptoms Questionnaire

|  | **Active**  **RS-tDCS**  **n (%)** | **Sham RS-tDCS**  **n (%)** |
| --- | --- | --- |
| Previous use of ≥1 preventive medications | 3 (30%) | 1 (8%) |
| Topamax | 0 | 1 (8%) |
| Depakote | 0 | 1 (8%) |
| Gabapentin | 1 (10%) | 0 |
| Antidepressant (unspecified) | 2 (20%) | 1 (8%) |
| TCA (unspecified) | 1 (10%) | 0 |
| Verapamil | 0 | 0 |
| Current use of ≥1 preventive medications | 4 (40%) | 5 (42%) |
| Topamax | 1 (10%) | 0 |
| Depakote | 0 | 0 |
| Gabapentin | 0 | 1 (8%) |
| Sertraline | 2 (20%) | 2 (17%) |
| Duloxetine | 1 (10%) | 1 (8%) |
| Venlafaxine | 0 | 1 (8%) |
| Trazodone | 0 | 1 (8%) |
| Doxepin | 0 | 1 (8%) |
| Verapamil | 0 | 1 (8%) |
| Previous Botox treatment | 4 (40%) | 1 (8%) |
| Previous use alternative therapies | 7 (70%) | 5 (42%) |

**Supplemental Table 3**. Baseline preventative treatments

Note: Alternative therapies included massage therapy, herbal therapy, acupuncture/acupressure, biofeedback, and hypnosis.

**Supplemental Table 4**. Baseline Comorbidities

|  | **Active RS-tDCS Group** | **Sham RS-tDCS Group** |
| --- | --- | --- |
| **Any comorbidity** | 9 (90%) | 9 (75%) |
| **PTSD** | 9 (90%) | 8 (67%) |
| **Anxiety** | 2 (20%) | 5 (42%) |
| **Depression** | 2 (20%) | 5 (42%) |
| **ADHD/ADD** | 1 (10%) | 2 (17%) |
| **Adjustment Disorder** | 0 | 1 (8%) |

**Supplemental Figure 1.** Detection of outliers.


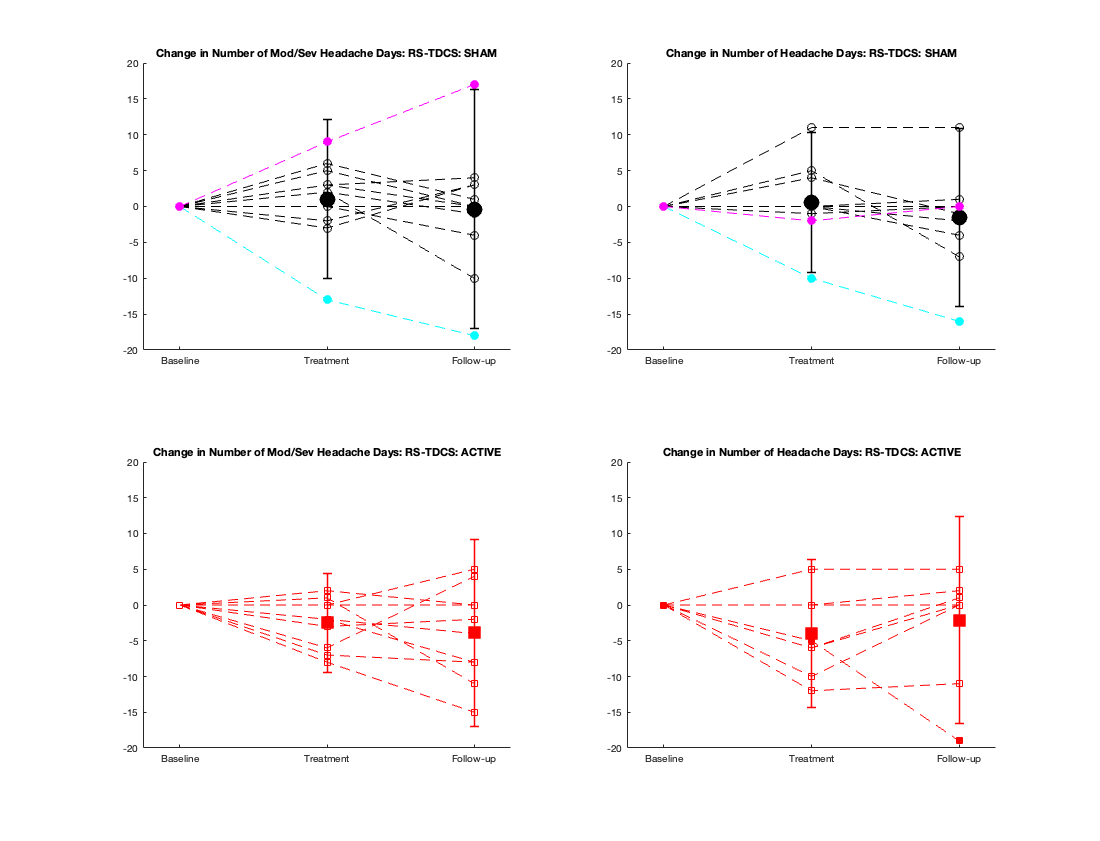


Outliers within each group were identified as individuals with values exceeding ±2.5 SD (error bars) at either timepoint for both primary and secondary outcome measures. Two SHAM participants (cyan and magenta) were identified as outliers on change in moderate-to-severe headache days. However, only one (cyan) was also an outlier on total number of headache days and therefore excluded. No participant in the active group was identified as an outlier on both measures.

**Supplemental Figure 2**. Treatment side effects reported by each group.


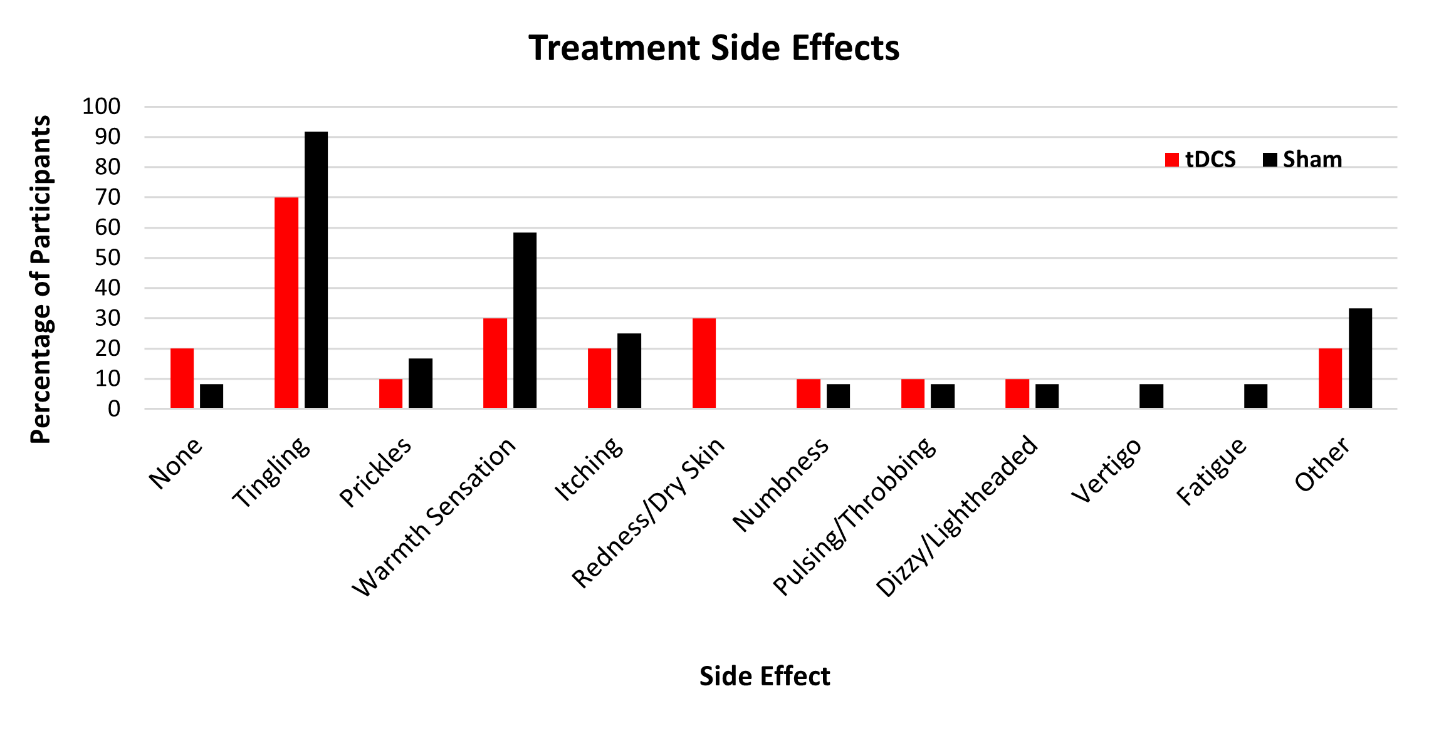

Supplement: Supplementary file 1 [file Data_Sheet_1.docx]
